# Supplementary material for: A Fatty Diet Induces a Jejunal Ketogenesis Which Inhibits Local SGLT1-Based Glucose Transport via an Acetylation Mechanism—Results from a Randomized Cross-Over Study between Iso-Caloric High-Fat versus High-Carbohydrate Diets in Healthy Volunteers
Source: Nutrients. 2022 May 7;14(9):1961. doi: 10.3390/nu14091961 (PMC9100393; doi:10.3390/nu14091961)
Supplement: Supplementary file 1 [file nutrients-14-01961-s001.zip › Supplementary Files S1. ClinicalTrials.gov (CT02088853) .pdf]

Protocol Registration Receipt
03/14/2014

Diet Induced Intestinal Mucosal Adaptation

This study is currently recruiting participants.
Verified by Prof Lars Fandriks, Göteborg University, March 2014

Sponsor:	Göteborg University
Collaborators:	Sahlgrenska University Hospital, Sweden
Information provided by (Responsible Party):	Prof Lars Fandriks, Göteborg University
ClinicalTrials.gov Identifier:	NCT02088853

Purpose

Human beings are 'omnivores' meaning that all principal components of food (i.e. the macronutrients: carbohydrates, fat, proteins) can be assimilated by the gastrointestinal tract. When the gut mucosa is exposed to dietary changes it adjusts its functional behaviour. For example, a fatty diet demands certain digestive mechanisms, whereas others are needed to take care of a carbohydrate rich diet. Such dietary induced changes in appearance and functionality of the small intestinal mucosa have been described in animals but only little is known about it in man. The present project aims at elucidating in man if a 2 weeks diet dominated by either fat or carbohydrates, but with similar energy content, is associated with changes in the small intestinal mucosal appearance and metabolic signalling capacity.

Condition	Intervention	Phase
Healthy Conditions	Dietary Supplement: high fat diet (hfd)<br>Dietary Supplement: high carb diet	N/A

Study Type: Interventional
Study Design: Basic Science, Crossover Assignment, Open Label, Randomized, N/A
Official Title: Small Intestinal Adaptation to Isocaloric Diets Dominated Either by Fats or Carbohydrates

Further study details as provided by Prof Lars Fandriks, Göteborg University:

Primary Outcome Measure:

- Mucosal surface enlargement factor [Time Frame: Appearance after 2 weeks of diet] [Designated as safety issue: No]

  The enlargement of the luminal surface area due to the villi structure is a morphometric factor defined as the total area of the mucosal surface in relation to the relatively flat area of the muscular is mucosae.

Secondary Outcome Measures:

- Epithelial electrical current in-vitro [Time Frame: The condition after 2 weeks diet] [Designated as safety issue: No]

  Mucosal biopsies are mounted in minUssing chambers. Ion-transporting capacity via SGLT-1 transported is reflected when switching from glucose-free to glucose-containing luminal solution, selectivity of transporter is confirmed by addition of phloridzin.

- Mucosal electrical resistance in vitro [Time Frame: The condition after 2 weeks diet] [Designated as safety issue: No]

  Mucosal biopsies are mounted in miniUssing chambers. Electrical resistance reflecting paracellular permeability is assessed when switching from glucose-free to glucose-containing luminal solution.

- Glycemic control following a test meal [Time Frame: The condition after 2 weeks diet] [Designated as safety issue: No]

  Blood concentrations of glucose and insulin are assessed over 2 hours following a mixed test meal.

Estimated Enrollment: 20
Study Start Date: March 2014
Estimated Study Completion Date: December 2014
Estimated Primary Completion Date: December 2014

Arms	Assigned Interventions
Experimental: high fat diet	Dietary Supplement: high fat diet (hfd)<br>Sixty % of the energy content is based on fat.
Active Comparator: high carb diet	Dietary Supplement: high carb diet<br>Sixty % of the energy content is based on carbohydrates.

Eligibility

Ages Eligible for Study: 18 Years to 65 Years
Genders Eligible for Study: Both
Accepts healthy volunteers.

Inclusion Criteria:

healthy volunteer not taking prescribed medications BMI ≤25 kg/m2

Exclusion Criteria:

BMI ≥26 kg/m2 smoker previous or current gastrointestinal disease significant abdominal surgery pregnancy/breast feeding drug intolerance of importance (particularly opiates and midazolam used during endoscopy) history of drug addiction

Contacts and Locations

Contacts

Lars Fändriks, MD, PhD	+46313424123	lars.fandriks@gastro.gu.se
Niclas Björnfot, RN	+46313428404	gastlab.su@vgregion.se

Locations

Sweden

Dept of Gastrosurgical R&E, Sahlgrenska Universityhospital — Recruiting

Gothenburg, Sweden, SE41345

Contact: Lars Fändriks, MD, PhD +46313424123

Principal Investigator: Lars Fändriks, MD, PhD

Investigators

Principal Investigator: Lars Fändriks, MD, PhD, Professor — University of Gothenburg

More Information

Responsible Party: Prof Lars Fandriks, Professor, MD, PhD, Göteborg University

Study ID Numbers: BUTTER

Health Authority: Sweden: Central Ethical Review Board, Stockholm, Sweden

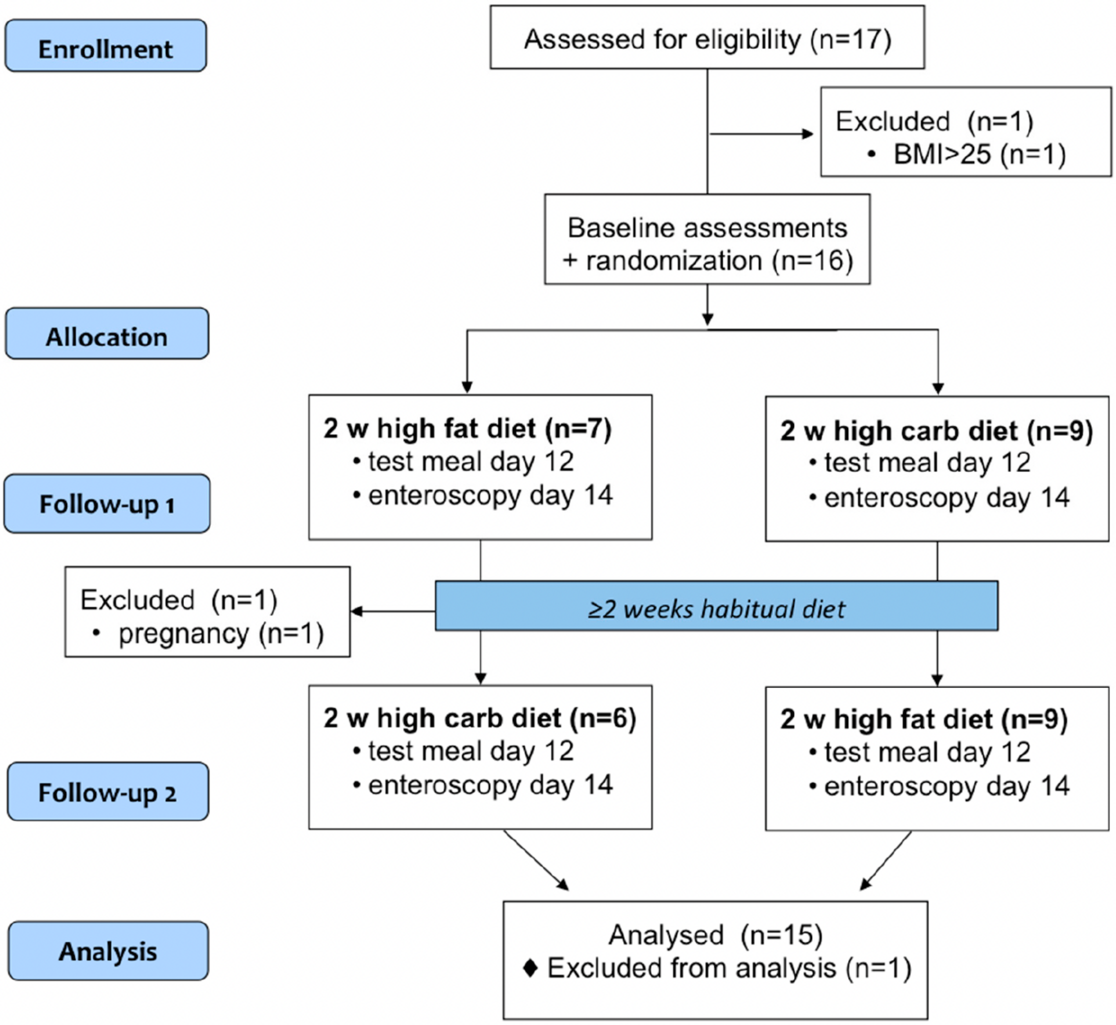

CONSORT diagram

Supplementary table

CONSORT 2010 checklist of information to include when reporting a randomised trial*

Section/Topic	Item No	Checklist item *(Butter II)*	Reported on page N
**Title and abstract**			
	1a	Identification as a randomised trial in the title	1
	1b	Structured summary of trial design, methods, results, and conclusions (for specific guidance see CONSORT for abstracts)	3 (in part)
**Introduction**			
Background and objectives	2a	Scientific background and explanation of rationale	6
	2b	Specific objectives or hypotheses	6
**Methods**			
Trial design	3a	Description of trial design (such as parallel, factorial) including allocation ratio	8
	3b	Important changes to methods after trial commencement (such as eligibility criteria), with reasons	-
Participants	4a	Eligibility criteria for participants	8
	4b	Settings and locations where the data were collected	8
Interventions	5	The interventions for each group with sufficient details to allow replication, including how and when they were actually administered	8
Outcomes	6a	Completely defined pre-specified primary and secondary outcome measures, including how and when they were assessed	8
	6b	Any changes to trial outcomes after the trial commenced, with reasons	-
Sample size	7a	How sample size was determined	8

	7b	When applicable, explanation of any interim analyses and stopping guidelines	-
Randomisation:			
Sequence generation	8a	Method used to generate the random allocation sequence	8
	8b	Type of randomisation; details of any restriction (such as blocking and block size)	8
Allocation concealment mechanism	9	Mechanism used to implement the random allocation sequence (such as sequentially numbered containers), describing any steps taken to conceal the sequence until interventions were assigned	8
Implementation	10	Who generated the random allocation sequence, who enrolled participants, and who assigned participants to interventions	8
Blinding	11a	If done, who was blinded after assignment to interventions (for example, participants, care providers, those assessing outcomes) and how	-
	11b	If relevant, description of the similarity of interventions	-
Statistical methods	12a	Statistical methods used to compare groups for primary and secondary outcomes	-
	12b	Methods for additional analyses, such as subgroup analyses and adjusted analyses	10
**Results**			
Participant flow (a diagram is strongly recommended)	13a	For each group, the numbers of participants who were randomly assigned, received intended treatment, and were analysed for the primary outcome	Suppl Fig 1
	13b	For each group, losses and exclusions after randomisation, together with reasons	Suppl Fig 1
Recruitment	14a	Dates defining the periods of recruitment and follow-up	-
	14b	Why the trial ended or was stopped	-
Baseline data	15	A table showing baseline demographic and clinical characteristics for each group	In ref 7
Numbers analysed	16	For each group, number of participants (denominator) included in each analysis and whether the analysis was by original assigned groups	11
Outcomes and estimation	17a	For each primary and secondary outcome, results for each group, and the estimated effect size and its precision (such as 95% confidence interval)	-
	17b	For binary outcomes, presentation of both absolute and relative effect sizes is recommended	In ref 7

Ancillary analyses	18	Results of any other analyses performed, including subgroup analyses and adjusted analyses, distinguishing pre-specified from exploratory	11 ff
Harms	19	All important harms or unintended effects in each group (for specific guidance see CONSORT for harms)	unknown pregnancy
**Discussion**			
Limitations	20	Trial limitations, addressing sources of potential bias, imprecision, and, if relevant, multiplicity of analyses	18
Generalisability	21	Generalisability (external validity, applicability) of the trial findings	18
Interpretation	22	Interpretation consistent with results, balancing benefits and harms, and considering other relevant evidence	18 (novel !)
**Other information**			
Registration	23	Registration number and name of trial registry	4
Protocol	24	Where the full trial protocol can be accessed, if available	From PI
Funding	25	Sources of funding and other support (such as supply of drugs), role of funders	3

*We strongly recommend reading this statement in conjunction with the CONSORT 2010 Explanation and Elaboration for important clarifications on all the items. If relevant, we also recommend reading CONSORT extensions for cluster randomised trials, non-inferiority and equivalence trials, non-pharmacological treatments, herbal interventions, and pragmatic trials. Additional extensions are forthcoming: for those and for up to date references relevant to this checklist, see www.consort-statement.org.
