# Supplementary material for: A Fatty Diet Induces a Jejunal Ketogenesis Which Inhibits Local SGLT1-Based Glucose Transport via an Acetylation Mechanism—Results from a Randomized Cross-Over Study between Iso-Caloric High-Fat versus High-Carbohydrate Diets in Healthy Volunteers
Source: Nutrients. 2022 May 7;14(9):1961. doi: 10.3390/nu14091961 (PMC9100393; doi:10.3390/nu14091961)
Supplement: Supplementary file 1 [file nutrients-14-01961-s001.zip › Supplementatory Files S2. Figures, texts, table.pdf]

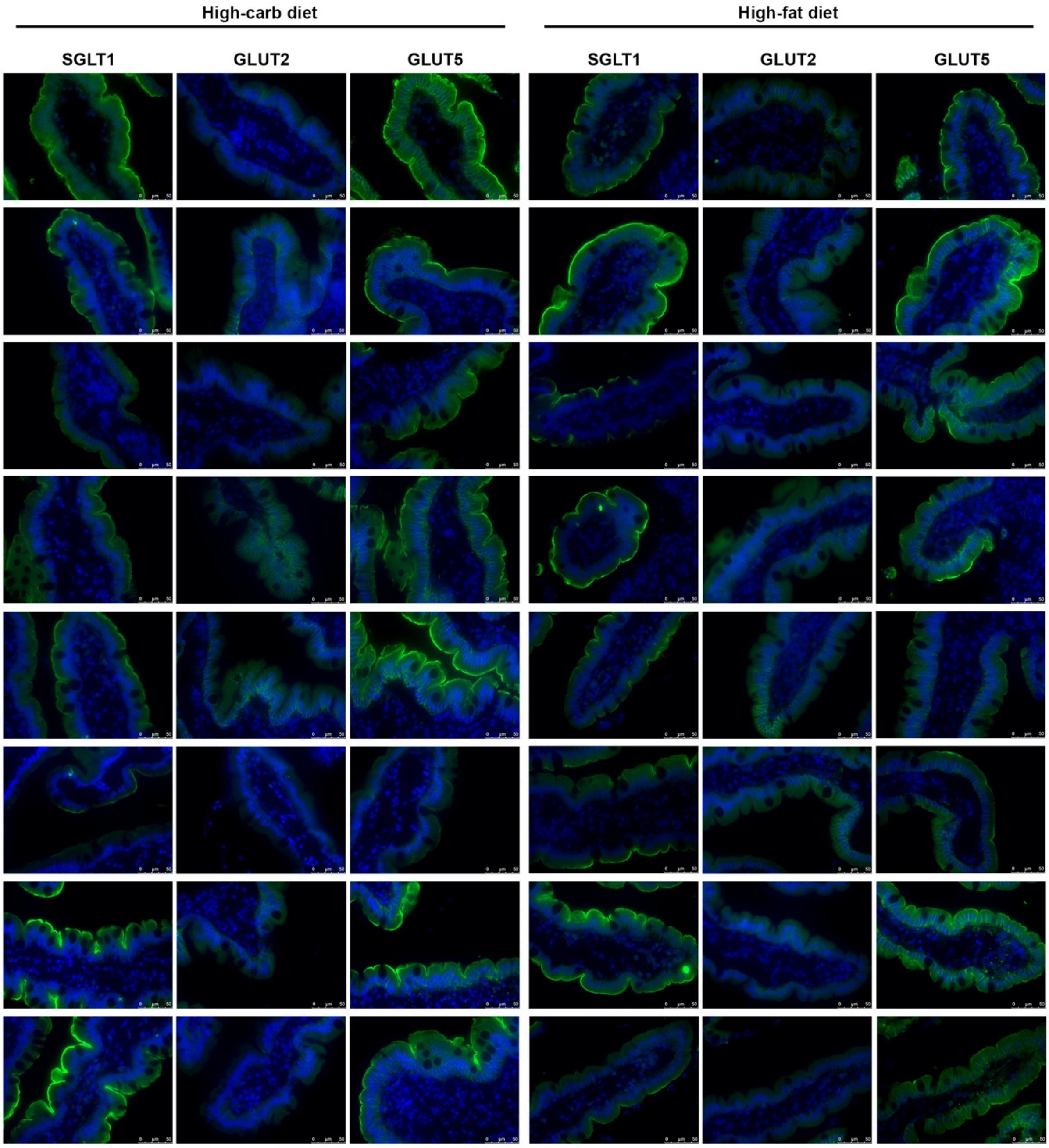

Supplementary Figure S1. The successful pairwise presentations (n=8) immunofluorescence stainings with SGLT1, GLUT2 and GLUT5 after high-carbohydrate and high-fat diets.

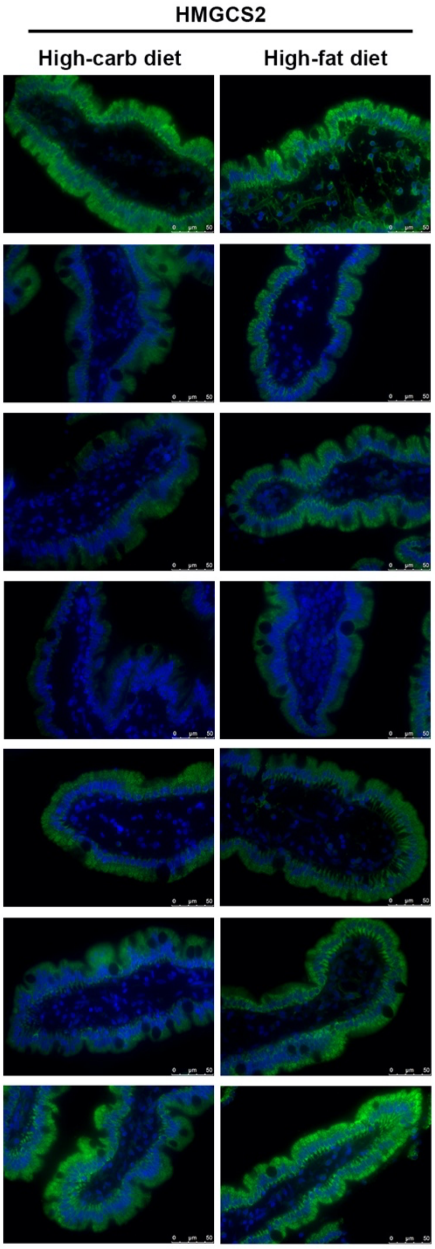

Supplementary Figure S2. Immunofluorescence of the successful pairwise stainings (n=7) of HMGCS2 after high- carbohydrate and high-fat diets.

Supplementary Text S1: Histology and immunofluorescence

The biopsies were chemically fixed in phosphate-buffered 4% formaldehyde, dehydrated, embedded in paraffin and cut in 5 µm sections. For immunofluorescence evaluation, the sections were rehydrated, antigens retrieved by boiling for 20 min in 10 mM citrate buffer (pH 6.0), blocked in 5% normal goat serum and incubated in primary antibody overnight at 4°C. After primary antibody incubation, slides were washed and incubated with secondary antibody for two hours in darkness at room temperature. After washing, slides were counter-stained with Hoechst staining and cover-slipped with ProLong Gold anti-fade reagent (Invitrogen, Carlsbad, CA, USA). Blocking buffer instead of primary antibody was used as negative control. For detailed list of primary and secondary antibodies used, see Supplementary table 1. The slides were analysed using a fluorescence microscope (Leica Microsystems, Wetzlar, Germany).

Supplementary Text S2: Protein extraction and western blot analyses

For frozen human jejunal mucosa specimens, tissue samples were sonicated in ice-cold protein extraction buffer (10 mM potassium phosphate (Sigma-Aldrich, St. Louis, MO, USA) pH 6.8, containing 1 mM ethylenediaminetetraacetic acid (Sigma-Aldrich), 10 mM 3-[(3-cholamidopropyl)dimethylammonio]-1-propanesulfonate (Boehringer Mannheim, Mannheim, Germany) and Complete protease inhibitor cocktail (Roche, Basel, Switzerland)). For Caco-2 and human enteroid cultures (see later for experiment details), cells on each semi-permeable membrane were scraped off in ice-cold RIPA buffer (Sigma-Aldrich) containing Complete protease inhibitor cocktail to detach cells from the membrane. They were then incubated on ice for 30 min, and the Caco-2 cultures were also sonicated. The homogenates were centrifuged (10,000 g, 10 min, 4°C) and the supernatant protein content quantified with the standard Bradford (Bio-Rad, Hercules, CA, USA) method. For western blotting of human biopsies and Caco-2 cultures, homogenates were diluted in SDS buffer (Invitrogen, Carlsbad, CA, USA) and heated at 70℃ for 10 min before loading on NuPage 10% Bis-Tris gels (Invitrogen). Electrophoresis was run using MOPS buffer (Invitrogen). For western blotting of the enteroid samples, homogenates were diluted in Laemmli buffer (Bio-Rad) and heated at 95˚C for 5 min

before loading on Criterion TGX Stain-Free Precast gels (Bio-Rad) and electrophoresis was run using tris/glycince/SDS buffer (Bio-Rad). After the electrophoresis, proteins were transferred to polyvinyldifluoride membranes (Amersham, Buckinghamshire, UK or Bio-Rad) using iBlot (Invitrogen) or Trans-Blot Turbo (Bio-Rad) blotting systems. Membranes were incubated with primary antibody followed by secondary antibody (see Supplementary table 1 for specific antibodies used). Chemiluminescense was developed with addition of CDP Star (Tropix, Bedford, MA, USA), WesternBright Quantum/Sirius (Advansta, Menlo Park, CA, USA) or Clarity Max Western ECL Substrate (Bio-Rad). Images were captured by the ChemiDoc system (Bio-Rad), and semi-quantification was performed using Quantity One (Bio-Rad) or Image Lab (Bio-Rad) software. Membranes were sequentially stripped using ReBlot Plus Mild (Merck Millipore, Burlington, MA, USA). Glyceraldehyde 3-phosphate dehydrogenase (GAPDH) was used as control for equal loading for each tested sample.

Supplementary Text S3: Ussing chamber experiments

Jejunal biopsies were immediately immersed in ice-cold oxygenated (95% O_2 and 5% CO_2) Krebs solution with the following composition (in mM): 118 NaCl, 4.69 KCl, 2.52 $CaCl_2$, 1.16 $MgSO_4$, 1.01 NaH_2PO_4, 25.0 $NaHCO_3$, and 11.1 glucose or mannitol (when glucose-free). Biopsies were then mounted in mini-Ussing chambers that had an insert with diameter of 2 mm, giving an area of 0.034 cm^2 (Warner instruments, Hamden, CT, USA). After mounting, each half chamber was filled with 5 mL Krebs solution, bathing both the mucosal and serosal sides of the specimen. The solution was maintained at 37°C and continuously oxygenated with 95% O_2 and 5% CO_2 and stirred by gas flow in the chambers. Initially the Krebs solution on the luminal side was held glucose-free to allow study of the electrogenic response to addition of glucose. The potential difference (PD) was measured with a pair of matched calomel electrodes (REF401, Radiometer analytical, Copenhagen, Denmark). The square wave pulse method was used to determine the tissue's epithelial electrical resistance (R_{ep}). The epithelial net ion current (I_{ep}) was obtained using Ohm's law (I_{ep}= PD/R_{ep}). Briefly, the method is based on the concept that the epithelium acts as a capacitor and resistor coupled in parallel. Short current pulses charge the epithelial capacitor, and when the current ends the capacitor is gradually discharged.

The epithelial voltage response, as assessed from the discharge curve, and the magnitude of the applied current were used for calculation of R_{ep}. Data were collected using an amplifier and specially constructed software developed in LabView (National Instruments, Austin, TX, USA).

Supplementary Text S4: Caco-2 and human jejunal enteroid monolayer cell cultures

Caco-2 cells at passage 48 to 52 **(**Sigma-Aldrich, St. Louis, MO, USA**)** were seeded onto 12-well transwell membranes (3.0 µm pore size, Corning, Corning, NY, USA) and expanded in expansion media (Dulbecco's Modified Eagle Medium (DMEM, 25 mM glucose, Invitrogen), with 10% fetal bovine serum (Invitrogen), 1X non-essential amino acids (NEAA, Invitrogen), 1X penicillin-streptomycin (PEST, Invitrogen)) on both sides the membrane until confluency. When confluent, cells differentiated into small intestine-like monolayers by being cultured in high glucose, serum free (SF) media (high glucose DMEM, 1X NEAA, 1X PEST) on the apical side and insulin-transferrin-selenium (ITS) media (high glucose DMEM, 1X ITS (Invitrogen), 1X NEAA, 1X PEST) on the basolateral side of the membrane for 14 days. Transepithelial electrical resistance (TEER) was measured using a voltmeter (Merck Millipore) during differentiation.

The human jejunal enteroid culture was established from endoscopic biopsies taken from the jejunal portion of the small intestine from one healthy volunteer (23 years old, female, ethics application number 049-16) using the technique developed by Sato *et al.(Gastroenterology 2011;141(5):1762-72).* In short, crypts were isolated, seeded in Matrigel (Corning) in 24-well plate and cultured with Wnt3A-supplemented expansion media (IntestiCult Organoid Growth Media (STEMCELLS, Vancouver, Canada), 10 µM Y-27632 (Sigma-Aldrich), 0.1% gentamycin (Invitrogen)) in order to enrich stem cells. For passage, Matrigel encapsulated cultures were treated with ice-cold Cell Recovery Solution (Corning) on ice for 1 hour before being centrifuged and washed. Cystic cultures were broken into smaller fragments by pipetting, before seeded into Matrigel again. Human jejunal enteroid cultures at passage 12 to 14 were seeded onto collagen-IV coated (15 µg/cm^2, Sigma-Aldrich) 24-well transwell membranes (0.4 µm pore size, Corning) and expanded in expansion media on both sides of membrane until confluent. When confluent, medium was exchanged to differentiation media (1:1 IntestiCult

Organoid Growth Media component A: DMEM/F12 with GlutaMAX (Invitrogen) and 15 mM HEPES (Invitrogen), 0.1% gentamycin (Invitrogen)) for 7 days. TEER was measured using during differentiation. Some membrane cultures were fixed in Karnowsky's fixative overnight, as previously described [1], for morphological analysis using transmission electron microscopy. Differentiated monolayers of Caco-2 cells and small intestine-like human enteroids were cultured for 48 h in low concentration glucose (5.5 mM) media (SF and ITS for Caco-2, and DMEM for enteroids). To study the ketogenesis in the differentiated monolayers, different combinations of following additives were given to low glucose media: the short-chain fatty acid butyrate (10 mM, Sigma-Aldrich), the HMGCS2 inhibitor hymeglusin (1 μM for Caco-2 and 10 μM for enteroids, Santa Cruz Biotechnology, Dallas, TX, USA), the sirtuin inhibitor nicotinamide (5 mM, Sigma-Aldrich) and the mevalonate pathway blocker simvastatin (1 μM, Caco-2 only, Sigma-Aldrich). Hymeglusin and simvastatin were dissolved in dimethyl sulfoxide (DMSO, Sigma-Aldrich) and, therefore, DMSO was added to the rest of the groups to eliminate potential solvent effects. After 48 hours the basolateral media were collected and βHB concentration quantified according to a β-Hydroxybutyrate Colorimetric Assay Kit (Cayman Chemicals, Ann Arbor, MI, USA) and proteins were extracted from cultures for western blot analysis as previously described.

Supplementary Table S1: Antibodies

Primary antibody	Manufacturer	Catalog number	Application	Dilution
Anti-GAPDH	Novus Biologicals	NB100-56875	Western blot	1:500
	Imgenex	IMG-5143A	Western blot	1:500
Anti-GLUT1	Abcam	ab32551	Western blot	1:1000
Anti-GLUT2	Abcam	ab85715	Western blot	1:500
		ab192599	Western blot	1:1000
	Atlas Antibodies	HPA028997	Immunofluorescence	1:200
Anti-GLUT5	Atlas Antibodies	HPA005449	Western blot	1:250
				1:600
			Immunofluorescence	1:200
Anti-GPR40	Invitrogen	PA5-27148	Western blot	1:1000
Anti-GPR41	Invitrogen	PA5-75521	Western blot	1:500
Anti-GPR43	Millipore	ABC299	Western blot	1:500
Anti-GPR109A	Invitrogen	PA5-90579	Western blot	1:500
Anti-H3K9ac	Cell Signaling	9649	Western blot	1:1000
Anti-HMGCS2	Santa Cruz Biotechnology	sc-33828	Western blot	1:800
		sc-393256	Immunofluorescence	1:150
Anti-SGLT1	Abcam	ab14686	Western blot	1:500
	Atlas Antibodies	HPA055106	Immunofluorescence	1:100
Anti-SGLT2	Invitrogen	PA5-75567	Western blot	1:500
Anti-SGLT3	Proteintech	24327-1-AP	Western blot	1:2000
**Secondary antibody**	**Manufacturer**	**Catalog number**	**Application**	**Dilution**
Anti-mouse IgG AP	Santa Cruz Biotechnology	sc-2008	Western blot	1:20000
Anti-rabbit IgG AP	Santa Cruz Biotechnology	sc-2007	Western blot	1:20000
Anti-mouse IgG HRP	Cell Signaling Technology	7076	Western blot	1:2000
			Western blot	1:2000
Anti-rabbit IgG HRP	Cell Signaling Technology	7074	Western blot	1:2000
			Western blot	1:2000
Anti-mouse IgG Alexa 488	Invitrogen	A11001	Immunofluorescence	1:2000
Anti-rabbit IgG Alexa 488	Invitrogen	A11008	Immunofluorescence	1:500
